# CONSUMPTION OF MINIMALLY PROCESSED AND ULTRA-PROCESSED FOODS AMONG STUDENTS FROM PUBLIC AND PRIVATE SCHOOLS

**DOI:** 10.1590/1984-0462/;2019;37;2;00010

**Published:** 2019-02-25

**Authors:** Camila Silva Ferreira, Dyene Aparecida Silva, Cristiana Araújo Gontijo, Ana Elisa Madalena Rinaldi

**Affiliations:** a Universidade Federal de Uberlândia, Uberlândia, MG, Brazil.

**Keywords:** Food consumption, Schoolchildren, Industrialized foods, Consumo alimentar, Escolares, Alimentos industrializados

## Abstract

**Objective:**

To compare and analyze the consumption of minimally processed and ultra-processed foods among students from public and private schools.

**Methods:**

Study conducted in Uberlândia, MG, with fifth-grade students from three private and six public schools, selected by stratified cluster sampling. We collected data on food consumption using the 24-hour recall. Foods were classified into four groups (G) according to extent and purpose of processing: fresh*/*minimally processed foods (G1) culinary ingredients (G2), processed foods (G3), and ultra-processed foods (G4). Total energy intake (kcal) of each group, amount of sugar (g), sodium (mg), and fiber (g) were quantified and compared according to administrative affiliation (private or public).

**Results:**

Percentage of total energy intake was: G1 - 52%; G2 - 12%; G3 - 5%; e G4 - 31%. Energy intake from G1 (53 vs. 47%), G2 (12 vs. 9%), and G3 (6.0 vs. 0.1%), and amount of sodium (3,293 vs. 2,724 mg) and fiber (23 vs. 18 g) were higher among students from public schools. Energy intake from G4 (36 vs. 28%) and amount of sugar (20 vs. 14%) were higher among students from private schools. The consumption of foods from G1 in the school environment was higher among students from public schools (40 vs. 9%).

**Conclusions:**

Foods from G1 represent the highest percentage of total energy intake, while those from G4 constitute a third of calories consumed. Processed juice, sandwich cookie, processed cake, and breakfast cereals are more frequent among private school students; snacks and juice powder are more common for students from public schools.

## RESUMO

**Objetivo:**

Comparar e analisar o consumo de alimentos minimamente processados e ultraprocessados entre escolares das redes pública e privada.

**Métodos:**

Estudo realizado em Uberlândia, MG, com escolares do quinto ano do ensino fundamental em nove escolas (três privadas e seis públicas), selecionados por amostragem estratificada por conglomerado. O consumo alimentar foi analisado utilizando recordatório de 24 horas. Os alimentos foram classificados segundo extensão e propósito do seu processamento em quatro grupos (G): alimentos *in natura/*minimamente processados (G1), ingredientes culinários (G2), alimentos processados (G3) e ultraprocessados (G4). Os valores energéticos totais (kcal) provenientes de cada grupo, quantidade de açúcar (g), sódio (mg) e fibras (g) foram quantificados e comparados segundo dependência administrativa.

**Resultados:**

O consumo de energia foi: G1, 52%; G2, 12%; G3, 5%; e G4, 31%. Os valores energéticos provenientes de G1 (53 *vs.* 47%), G2 (12 *vs.* 9%) e G3 (6,0 *vs.* 0,1%), a quantidade de sódio (3.293 *vs.* 2.724 mg) e a de fibras (23 *vs.* 18 g) foram superiores em escolares da rede pública. O valor percentual energético do G4 (36 *vs.* 28%) e a quantidade de açúcar (20 *vs.* 14%) foram superiores em escolares da rede privada. O consumo do G1 na escola foi superior nos escolares da rede pública (40 *vs.* 9%).

**Conclusões:**

Alimentos do G1 representam o maior percentual do valor energético total e do G4, um terço das calorias ingeridas. Suco pronto, biscoito recheado, bolo industrializado, cereais matinais são mais frequentes em escolares da rede privada e salgadinhos e suco em pó nos da rede pública.

## INTRODUCTION

The dietary patterns of the world population are changing, mainly due to the higher consumption of ready-to-eat foods, which have high levels of fat and sugar, and lower intake of unprocessed foods, such as fruits, vegetables, tubers, and cereals.^1^ Data analysis of the dietary patterns of Brazilian adults identified that 69.5% of the total daily calories consumed come from fresh or minimally processed foods, 9.0% from processed, and 21.5% from ultra-processed.^2^

These dietary patterns observed in adults are also found in studies with Brazilian children and adolescents, who showed low intake of milk, fruits, and vegetables^3^ and high consumption of foods rich in sugar,^3^ ^,^ ^4^ fat,^3^ processed juices, and soft drinks.^3^ ^,^ ^4^ Results of a study carried out in Pelotas, RS, indicated that 19.7% of the energy intake in the diet of children younger than 24 months originated from ultra-processed products and this percentage was higher for those older than 24 months (36.1%).^5^ Among children aged two to ten years assisted at the Basic Health Unit (*Unidade Básica de Saúde* - UBS), the percentage of energy intake of ultra-processed products was 47.0%.^6^

The National Adolescent Student Health Survey (*Pesquisa Nacional de Saúde do Escolar* - PeNSE) analyzed the consumption of markers of healthy and non-healthy eating. According to data from the last PeNSE (2015), the regular intake (≥5 days/week) of beans was 60.7%; vegetables, 37.7%; fruits, 32.7%; croquettes, 13.7%; candies, 41.6%; soft drinks, 26.7%; and other processed/ultra-processed foods, 31.3%. With respect to food consumption in the school environment, only 38.1% of public school students reported consuming meals provided by the National School Feeding Program (*Programa Nacional de Alimentação Escolar* - PNAE). Regarding foods purchased in cafeterias or brought from home and consumed during school hours, the prevalence of soft drinks, candies, and processed snacks among public school students was 58.5, 49.7, and 63.7%, and for private school students, it was 70.6, 62.3, and 60.0%, respectively.^7^

Analysis of food intake among students from public and private schools and the prevalence of consumption in intra- and extra-school environments allow us to identify differences and similarities in these environments, as well as substantiate the elaboration of intervention strategies. Additionally, the dietary intake research based on the degree and extent of processing is consistent with the verification conducted with the Brazilian population and with the classification proposed by the Dietary Guidelines for the Brazilian Population.^8^ Thus, the objectives of this study were to compare and analyze the consumption of minimally processed, processed, and ultra-processed foods among students from public and private schools.

## METHOD

Cross-sectional study performed with fifth-grade students from private and municipal public schools in Uberlândia, MG, from March to August 2013. In that year, the city had 37 municipal schools with 4,192 students regularly enrolled and 73 private schools with 1,186 students. These schools were distributed in five geographic city areas (downtown, west, east, north, and south).

Students were chosen by stratified cluster sampling in two stages. The first stage consisted of selecting schools by stratified sampling, considering the city area and administrative affiliation (public and private) in proportion to the number of students; the second selected fifth-grade classes from each school. Sample size calculation considered 30 students per class upon prior consultation with the schools. The final estimated sample was 270 fifth-grade students distributed in nine schools (six public and three private) at a ratio of 3:1, respectively, with a maximum error of 6%.

The total number of students enrolled in the selected classes was 272 (205 from public and 67 from private schools). However, due to some students refusing to participate, guardians not signing the informed consent form, and implausible total energy intake (≤400, and ≥7000 kcal),^9^ the final sample comprised 206 students, 151 from public and 55 from private schools (response rate=76%).

We collected data on food consumption using a 24-hour recall of a typical day of the week. Fifth-grade students were chosen precisely for their age group, as only around 10 to 12 years, the individual can give more reliable answers about food consumption.^10^

A pilot study previously tested the use of the instrument to estimate the time of application, collection logistics, and possible doubts. The 24-hour recall followed the five steps recommended by the United States Department of Agriculture, in order to improve quality and reliability of foods reported.^11^

We used photographic records^12^ and described quantities in a table with household measures^13^ to assist students in determining the amount of food consumed. The software Diet Pro 5i estimated the total energy intake (kcal) and amount of sugar (kcal), sodium (mg), and dietary fibers (g), using the Brazilian Food Composition Table^14^ as a reference, complemented by the United States Department of Agriculture Food Composition Databases.^15^ For meals and/or preparations which were not in the selected tables, we used the standardization of recipes proposed by Pinheiro^13^ and information on nutrition labels for ultra-processed foods.

Food classification based on the extent and purpose of processing followed the 2010 proposal by Monteiro and collaborators,^16^ updated in 2012.^17^ All foods reported in the 24-hour recall were categorized into the following groups:

- G1) Fresh or minimally processed foods;
- G2) oils, fats, salt, and sugar, or culinary ingredients;
- G3) processed foods;
- G4) ultra-processed foods.

We separated the ingredients of all culinary preparations (e.g., pasta with tomato sauce, meatball, rice and beans, polenta with minced meat, among others) to classify them in their respective food groups. All recipes were based on a table of preparation with household measures^13^ to adopt a standard and due to students not knowing the ingredients of the meals.

The analyses of food consumption focused on the description of total energy intake, percentage of total energy intake of each group (G1, G2, G3, and G4) based on the extent and purpose of food processing, percentage of total energy intake of sugar, and total amount of sodium (mg) and dietary fibers (g) consumed. We also analyzed the percentage of total energy intake in the school environment separately. Public school students answered questions on the consumption of meals provided by the Municipal School Feeding Program. All analyses considered the group of children from the sample and administrative affiliation of the school.

Categorical variables were expressed in relative frequencies. The Shapiro-Wilk test evaluated the normality of continuous variables. Both were described as median and 1st (1stQ) and 3rd quartiles (3rdQ). We used the Mann-Whitney test to analyze continuous variables according to administrative affiliation, and the chi-square test for categorical variables. All observations were corrected by the design effect (clustering) using the weighting factor (sampling weight). We adopted a significance level of 5%. The software Epi-Info^®^ version 3.5.2 analyzed the data. The Research Ethics Committee of Universidade Federal de Uberlândia approved this study - report no. 461540/Certificate of Presentation for Ethical Consideration (*Certificado de Apresentação para Apreciação Ética* - CAAE): 06257813.70000.5152.

## RESULTS

This study comprised 206 students: 151 (78%) from public and 55 (22%) from private schools. Most were females (53%), with no difference regarding administrative affiliation (p=0.69). The median 1stQ-3rdQ age of public school students was superior to that of private school students [10.6 (1stQ=10.2; 3rdQ=11.2) vs. 10.2 years (1stQ=10.0; 3rdQ=10.5)] (p<0.001).

Considering the value and percentage of energy intake according to food groups, G1 (52%) is predominant among all students, followed by G4 (31%), G2 (12%), and G3 (5%). With respect to administrative affiliation, the total energy intake by students from public schools was superior to those from private schools. The value and percentage of energy intake of foods from G1, G2, and G3 were higher for public school students, while for G4, the numbers were superior among students from private schools. Sugar intake was greater among private school students, and sodium and dietary fibers were higher in those from public school (Table 1).

**Table 1:** Total energy intake and percentage of energy intake of each food group, percentage of energy intake of sugar, and amount of sodium and fiber consumed by students from public and private schools. Uberlândia, MG, 2013 (n=206).

	Total	Public	Private	p-value*
TEI (kcal)	1943 (1555;2439)	2010 (1555;2476)	1802 (1524;2031)	0.01
Group 1 (kcal)	934 (748;1227)	989 (755;1274)	838 (696;985)	<0.001
Group 1 (%)	52 (44;60)	53 (45;62)	47 (40;54)	<0.001
Group 2 (kcal)	198 (146;275)	207 (162;288)	144 (110;235)	<0.001
Group 2 (%)	12 (8;14)	12 (9;14)	9 (6;13)	<0.001
Group 3 (kcal)	84 (0;153)	147 (1;153)	2 (0;135)	<0.001
Group 3 (%)	5 (0;9)	6 (0;10)	0.1 (0;6)	<0.001
Group 4 (kcal)	569 (316;860)	544 (293;817)	656 (455;1010)	<0.001
Group 4 (%)	31 (19;42)	28 (18;40)	36 (28;46)	<0.001
Sugar (kcal)	290 (153;532)	285 (136;492)	333 (206;621)	<0.001
Sugar (%)	15 (9;24)	14 (8;22)	20 (13;27)	<0.001
Sodium (mg)	3107 (2537;4084)	3293 (2632;4234)	2724 (2254;3392)	<0.001
Fiber (g)	21 (16;29)	23 (17;31)	18 (12;22)	<0.001

Values expressed as median (1st quartile; 3rd quartile); *for this analysis, we used the Mann-Whitney test; Group 1: fresh or minimally processed foods; Group 2: culinary ingredients; Group 3: processed foods; Group 4: ultra-processed foods; TEI: total energy intake; kcal: kilocalories; mg: milligrams; g: grams.

Table 2 lists the foods reported by students. Regarding foods from G1, the consumption of rice and meat was predominant for all students. Intake of milk, vegetables, fruits, flour, and eggs was greater among private school students, while consumption of beans, pasta, and coffee was higher among the ones from public schools. Consumption of culinary ingredients (G2) differed only for butter, more consumed by public school students. The profile of foods from G4 was distinct between students from public and private schools. Private school students consumed more products such as processed juice, sandwich cookie, milk-based sugary drinks, breakfast cereals, processed cakes, and processed meat products (Table 2).

**Table 2:** Percentage of food consumed by students according to food groups and type of school. Uberlândia, MG, 2013 (n=206).

Food	Public %	Private %	p-value^#^
Fresh or minimally processed foods (G1)			
Rice	96	94	0.48
Meat	95	93	0.25
Beans	89	69	<0.001
Vegetables	68	85	<0.001
Milk	62	73	0.02
Fruits	56	76	<0.001
Pasta	37	11	<0.001
Coffee	31	9	<0.001
Roots and tubers	24	18	0.19
Eggs	23	34	0.01
Flour	18	33	<0.001
Other*	7	9	0.51
Culinary ingredients (G2)			
Vegetable oil	99	100	0.37
Table salt	99	100	0.37
Sugar	53	60	0.17
Butter	16	5	<0.001
Olive oil	3	5	0.26
Processed foods (G3)			
Bread	65	51	<0.001
Canned and preserved foods	36	34	0.80
Cheese	26	36	0.02
Ultra-processed foods (G4)			
Candies	59	51	0.11
Soft drinks	47	40	0.17
Juice powder	45	13	<0.001
Chocolate powder	42	51	0.07
Margarine	40	44	0.52
Cold cuts	28	34	0.20
Processed juice	20	36	<0.001
Cakes and cookies	19	16	0.48
Processed snacks and popcorn	17	7	0.01
Sandwich cookie	16	27	<0.001
Cracker	11	16	0.13
Bread	9	22	<0.001
Milk-based sugary drinks	9	18	<0.001
Breakfast cereal and granola bar	5	14	<0.001
Condensed milk	5	5	0.71
Cream	4	13	<0.001
Croquettes, salty pastries, and French fries	3	25	<0.001
Processed muffin	3	13	<0.001
Processed meat products**	3	13	<0.001
Mayonnaise	2	11	<0.001
Other***	7	7	0.80

^#^Chi-square test; *other cereals (corn and oat), oilseeds, honey, and teas; **bacon, hamburger, and nuggets; ***ketchup, sweetener, and instant noodles.

The percentage of students who reported consuming some type of food exclusively within the school environment was 74%. This consumption is more frequent in private schools than in public ones (93 vs. 69%; p<0.001). Only 40% of public school students declared consuming preparations provided by the Municipal School Feeding Program. The median energy intake, in kcal, in the school environment was higher among students from private schools compared to public ones [284 (1stQ=198; 3rdQ=361) vs. 170 (1stQ=0; 3rdQ=370); p<0.001], representing 15 to 9% of the total energetic intake, respectively (data not shown in tables).

After analyzing food consumption within the school environment in more detail, we found that the percentage of energy intake of foods from G1 was higher among students from public schools (23 vs. 7%; p<0.001). In G1, only the intake of fruit and fresh fruit juice within the school environment was superior among private school students (Table 3). The percentage of energy intake of foods from G4 was predominant in students from private schools (35 vs. 16%; p<0.001) (data not shown in tables).

**Table 3:** Percentage of food consumed by students in the school environment according to food groups and type of school. Uberlândia, MG, 2013 (n=206).

Item consumed	Public %	Private %	p-value^#^
Fresh or minimally processed foods (G1)	39	9	<0.001
Meat*	30	0	<0.001
Rice*	24	0	<0.001
Beans*	20	0	<0.001
Pasta*	11	0	<0.001
Vegetables*	9	0	<0.001
Eggs*	3	0	0.07
Milk	2	0	0.12
Fruit and fresh fruit juice	1	11	<0.001
Culinary ingredients (G2)	37	4	<0.001
Vegetable oil*	36	0	<0.001
Table salt*	36	0	<0.001
Sugar	6	4	0.32
Processed foods (G3)	17	9	<0.001
Tomato paste*	10	0	<0.001
Bread	6	7	0.22
Ultra-processed foods (G4)	45	89	0.36
Candies	20	7	<0.001
Processed juice	10	24	<0.001
Processed snacks	5	5	0.94
Croquettes and salty pastries	4	24	<0.001
Sandwich cookie	4	18	<0.001
Cakes and cookies	3	7	0.05
Soft drink	2	14	<0.001
Chocolate powder*	2	0	0.12
Processed muffin	1	11	<0.001
Milk-based sugary drinks	1	13	<0.001
Bread	0	2	<0.001
Margarine	0	2	<0.001
Other**	2	9	<0.001

^#^Chi-square test; *foods provided by the Municipal School Feeding Program; **cracker, granola bar, mortadella, and *requeijão* (Brazilian cream-cheese)

## DISCUSSION

The classification of foods and food products adopted in this study favors the analysis of food sources and way of processing. Analyzes focused only on nutrients are insufficient to demonstrate the relationship between the negative effects of processed and ultra-processed products.^18^ Moreover, this new approach is more comprehensive and enables targeting intervention strategies undertaken by health professionals.

The main results found in the present study were:

- Foods from G1 represent the highest percentage of daily energy intake among students from both private and public schools, followed by foods from G4, higher among private school students.
- The profile of fresh/minimally processed and ultra-processed foods consumed by students differ according to the type of school administration.
- Preparations provided by the Municipal School Feeding Program are a good alternative to potentially reduce the consumption of ultra-processed foods in the school environment and could contribute to increasing the percentage of daily energy intake of foods from G1.

In this study, the foods from G1 most consumed by students (rice, meat, and beans) are similar to those consumed by the Brazilian population.^16^ We identified a higher fiber intake among public school students, possibly due to the greater percentage of energy from the consumption of beans. A study suggests that non-habitual consumption of beans is a risk factor for insufficient dietary fiber intake.^19^

The purchase and consumption of some foods from G1, such as milk, fruits, and vegetables, tend to increase with a higher income.^16^ In the present study, we also observed a greater frequency of milk, fruits, and vegetables among private school students. However, this comparison should be made with caution since we did not analyze income.^20^

As for foods from G3, bread was one of the products with a higher incidence of consumption in this and other studies.^16^ ^,^ ^21^ Processed bread was more frequent among students from public schools and ultra-processed ones among those from private schools. Foods from G4 represented more than one-third of the energy consumed, a result similar to the pattern described for the Brazilian population.^2^ ^,^ ^16^ Although more than 30% of the total energy intake by students from private and public schools corresponded to foods from G4, there is a difference in the profile of foods consumed. Candies were the most consumed among students, with no significant difference between public and private schools.

In the study by Monteiro,^16^ all food products, except cookies and processed meat, were more consumed as income increased. In the present study, the products most often mentioned by public school students were juice powder, and processed snacks and popcorn. In private schools, there was a predominance of cheese, processed juice, sandwich cookie, croquettes, salty pastries, and French fries, breakfast cereal and granola bar, milk-based sugary drinks, processed meat products, and processed muffin. Despite our results being similar to those identified by Monteiro et al.,^16^ we emphasize that it is impossible to state that income can explain the difference in the profile of foods consumed by students from public and private schools.

Ultra-processed products frequently consumed within the school environment by students from private schools in different Brazilian regions were processed juice, croquettes and salty pastries, sandwich cookie, and soft drink.^22^ ^,^ ^23^ ^,^ ^24^ Although there is no recommendation on the adequate percentage of intake for these products, these values are clearly high, particularly when considering the excess sugar and sodium and low content of dietary fiber found in the foods consumed by students. The percentage of energy contribution in each food group can vary according to income, showing an inverse relationship with the intake of foods from G1 and G2 and a direct relationship from G3 and G4.^25^ However, a research conducted in Brazil shows that this contribution has a direct relationship with the group with fresh/minimally processed, processed, and ultra-processed foods, and an inverse relationship with culinary ingredients.^19^

Most ultra-processed products are semi-perishable and require no prior preparation for consumption, being also defined as “convenience foods” or “fast foods”.^26^ They are designed to be portable, practical, and accessible, inducing eating patterns such as skipping main meals, eating while doing other activities - e.g., watching television, driving a car, or working -, and eating alone,^26^ factors that mainly relate to overweight.^27^ Another relevant reason to stimulate the decrease in their consumption is the weakening of traditional food cultures and loss of culinary diversity.^27^

We identified that 40% of students consume meals provided by public schools. Sturion et al.^28^ revealed that 46% of students consumed the meal served by the school. These results are alarming since students prefer products of low nutritional quality.^28^ A study by Cruz et al.^29^ showed that 84.4% of students consumed school meals and 28% bought ultra-processed foods at cafeterias near the school. The availability of ultra-processed foods in the surroundings of public schools was superior to that of minimally processed products, favoring the consumption of ultra-processed foods in the school environment.^30^

We underline the analysis of food consumption in this age group aligned with the proposal by Monteiro^16^ and later with the Dietary Guidelines for the Brazilian Population^8^ as the main positive point of this study. This classification allowed us to consider, at the same time, food consumption having food and food combinations as the main object and, indirectly, the nutrients found in these foods. Additionally, this classification enables nutritional education programs to focus on foods and food preparations, while respecting the culture, local eating habits, and even rescuing the cuisine.

The limitations to be highlighted relate mainly to the application of a 24-hour recall for each student, the adoption of recipe standardization, and the sample number being lower than the one proposed by the sample size calculation, especially in public schools (82% in private and 73% in public schools). Despite the sample having 206 recalls, which contributed to reducing inter-individual variability, the application of more than one recall could decrease inter-individual variability even more. Concerning recipe standardization, we needed to adopt a model for the preparations reported by the students since they did not know the ingredients of all meals consumed. Standardization of amounts of oil and salt added to preparations can impact the results found, particularly because oil affects the energy intake. As we use default values, it is possible we underestimated oil and especially salt consumption.

The lack of maternal schooling and income data to verify the direct effect of socioeconomic factors on food consumption was also considered a limitation. These data were not collected since, in the pilot study, we found that students did not know this information, and the administration of the schools did not allow us to access it in the school records. Thus, we used the type of administrative affiliation as a proxy of these factors. We believe that this study represented different incomes due to the clustering of the sample by geographical city areas.

A final limitation identified was the time interval between data collection (2013) and its publication. Despite the four-year interval, we did not find changes in educational policies or school interventions that could alter the food intake among students in this period. We know that schools work the subject of school feeding in isolation, mainly on themed days (such as fruit day, and World Food Day). Data from PeNSE (2009, 2012, 2015) and the Household Budget Survey (HBS) (2002-2003 and 2008-2009) are still used in recent publications, even with time intervals exceeding five years.

Public school students show a higher percentage of energy intake of foods from G1, including in the school environment, possibly as a result of the preparations provided by the school feeding program. On the other hand, the percentage of foods from G4 was higher among students from private schools, both in the intra- and extra-school environment. Although the percentage of energy intake of foods from G4 among public school students was lower than for those from private schools, the consumption of these foods also represents a significant percentage of the daily energy intake for this population, with differences concentrated in the type of food consumed. Some ultra-processed foods consumed by students from public and private schools differed. Students from private schools consumed more processed juice, sandwich cookie, processed cake, breakfast cereals, and milk-based sugary drinks, while for public school students, the intake of snacks and juice powder was more frequent. In this scenario, developing strategies for nutrition education targeted at students from both private and public schools and their families is essential.
